# Disability, Offense, and the Expressivist Objection to Medical Aid in Dying

**DOI:** 10.1093/jmp/jhae031

**Published:** 2024-08-26

**Authors:** Brent M Kious

**Affiliations:** University of Utah, Salt Lake City, Utah, USA

**Keywords:** *disability*, *disrespect*, *medical aid in dying*, *offense*

## Abstract

One criticism of medical aid in dying (MAID) is the expressivist objection: MAID is morally wrong because it expresses judgments about disabilities or persons with disabilities, that are offensive, disrespectful, or discriminatory. The expressivist objection can be made at the level of individual patients, medical providers, or the state. The expressivist objection originated with selective abortion, and responses to it in that context typically claim either that selective abortion does not express specific judgments about disabilities, or that any judgments expressed are not offensive. This response is inadequate: MAID often does express negative judgments about disabilities, which could reasonably be seen as offensive. But, does this offensiveness make MAID wrong? Drawing on Joel Feinberg’s account of offense, I argue that it is unlikely that the offensiveness of the judgments expressed by individuals who seek MAID or through the state’s legalization of MAID is enough to make it morally impermissible.

## I. INTRODUCTION

A frequent criticism of medical aid in dying (MAID)—which can include physician-assisted suicide (or physician aid-in-dying) and voluntary euthanasia—is that it is in some sense offensive to persons with disabilities. Many who choose MAID do so because they are or will be afflicted by physical or mental impairments they regard as intolerable. Because these or very similar impairments are already borne by persons living—happily, meaningfully, productively—with disabilities, choosing MAID seems to express a pernicious form of disrespect for those persons, which is offensive to them. Some think this makes MAID morally wrong since it betrays pejorative judgments, held by any of a number of different parties, about the value of living with a disability or about the lives of persons with disabilities. This argument—what we can call the *expressivist objection* to MAID—might also be framed as saying that MAID involves an ableist bias, impugns the dignity of disabled persons, shows disrespect for them, or is discriminatory. I take these complaints to be interchangeable for present purposes.

In what follows, I hope to take a more nuanced approach to evaluating the expressivist objection than other critics have done: I argue that although the expressivist objection is initially plausible, it cannot bear the moral weight placed on it. Although MAID often does convey negative judgments about specific disabilities, these negative judgments, as well as the offense they occasion, do not typically make MAID morally impermissible, because the offense itself is not morally decisive.

To support this claim, I first describe and give examples of the expressivist objection, noting that it can be applied to the actions of the state, medical providers, or individual patients. I focus first on patient-level considerations. I review and reject a family of responses to the patient-level expressivist objection, all of which deny that MAID expresses anything offensive about disabilities. MAID, I claim, often really does express negative judgments about disabilities (though not always about *persons* with disabilities) and those expressions can be offensive. Then, however, I question how much this offensiveness should matter. I revisit Joel Feinberg’s (1985) account of offense and argue that his criteria for prohibiting offensive acts suggest that the offensiveness of MAID is unlikely to make it morally impermissible. I then examine state-level considerations. I assess whether plausible state-level obligations like neutrality with respect to different individuals’ conceptions of the good, showing equal regard for the claims of its citizens, or limiting especially pernicious forms of communication like hate speech, could support the claim that MAID legislation is, because of what it expresses, wrong. I conclude that they are not.

## II. THE EXPRESSIVIST OBJECTION

The expressivist objection is one of many criticisms that disability scholars have made of MAID, which range from concerns that such practices might lead persons with disabilities to feel pressure to die, to the thought that by permitting MAID, one is *perpetuating* ableism and making people more likely over time to think that being disabled is bad, to fears that MAID might erode social programs supporting persons with disabilities, or even that the state or medical providers might force euthanasia on unwilling disabled victims (a concern for which there is clear historical precedent). I do not consider these other criticisms here; they may be correct, and even decisive. Likewise, although MAID has been criticized because it represents a failure of imagination (the idea being that a disabling illness will not really be as bad as one supposes, if only because one will gradually habituate to it), and similarly because it may only be a reaction to unjustly limited social supports for persons with disabilities and their caregivers, I do not consider those objections here, either. In keeping with this narrowed scope, I assume that decisions to receive MAID are not merely the product of ignorance, limited imagination, or unjust and deplorable social conditions, but rather represent sustained, clear-eyed, and authentic assessments of the quality of one’s own life and of the value and disvalue of certain health states. This is not always true in real life, but it is a simplifying assumption that makes evaluating the expressivist objection itself easier.

Why focus on the expressivist objection, given all of the other things at stake? Although it is one objection that disability theorists might make for MAID, it may not be the most important one, and it is rarely made in isolation. Still, there are four reasons to address it specifically. First, it *is* commonly expressed in the bioethical literature—though often tangled up with other, potentially more compelling, objections, and not given quite as much philosophical precision as one would like. Second, it is important to inspect the expressivist objection separate from other disability-based arguments, because it is otherwise difficult to evaluate thoroughly any of them. Third, it parallels a similar, and more widely debated, objection to selective abortion. Fourth, and most importantly, I believe there is a widespread sense that we have strong and far-reaching obligations to avoid making and acting on, judgments of others that are critical of their personal characteristics, especially when these judgments pertain to characteristics with which they identify, even if our actions are nominally private or self-regarding. This is an assumption we should question, and the argument here is one step toward delimiting those obligations.

There are multiple versions of the expressivist objection, varying mainly with respect to who is engaged in the expressive act. One can find instances that are focused on what physicians express by providing MAID, on what the state expresses by legalizing it, and on what individual patients express. Consider, for example, the following statement from Carol Gill: “Doctors cannot comply with assisted suicide laws unless they agree that people whom they judge to be terminally ill have lives less worthy of full protection than other people’s life [sic].” (2010, 36). Here, the problem is that doctors betray a problematic judgment toward persons with disabilities when they engage in MAID. Others focus on expressions by the state. Daniel Sulmasy and colleagues write:

The chief reason that there is such widespread resistance to [physician-assisted suicide] in the disabled community [is]. . . that their dignity is deeply disrespected by the very fact that a society legally sanctions the notion that dependent persons like them can be considered better off dead. (2018, 1396)

Similarly, Scott Kim observes:

Imagine. . . someone who experiences suffering that is caused by a condition C. Suppose that such suffering is the right kind of suffering from the state’s perspective, i.e., suffering due to C is acceptable for EAS [(euthanasia and assisted suicide)]. But imagine persons with C who do not want EAS. Most likely, this will be the vast majority of persons with C. Such persons, from their perspective, might find this state-sanctioned criterion…discriminatory and an attack on their dignity, seeing it as a label that stigmatizes their lives as not worth living. (2022, 60)

Somewhat differently, Marilyn Golden and Tyler Zoanni focus on the expressions of individuals:

Fear, bias, and prejudice against disability play a significant role in assisted suicide. Who ends up using assisted suicide? . . . The overwhelming majority of the people in Oregon who have reportedly used that state’s assisted suicide law wanted to die not because of pain, but for reasons associated with disability, including the loss of dignity and the loss of control of bodily functions. . . . But as many thousands of people with disabilities who rely on personal assistance have learned, needing help is not undignified, and death is not better than reliance on assistance. Have we gotten to the point that we will abet suicides because people need help using the toilet? (2010, 17)

The expressions of different groups—individual patients, medical providers, and the state itself—may be subject to very different moral constraints. I begin by examining the expressive significance of the actions of private citizens who choose MAID (the *patient-level* objection). I then turn to state-level considerations, the validity of which may depend on what we find at the patient level: if the patient-level objection holds, it makes it more likely that the state-level objection holds, though there may be state-level considerations that do not depend on the patient level. Finally, I briefly examine the provider-level objection, though I see this as largely settled by patient-level and state-level considerations.

## III. DOES MAID INVOLVE OFFENSIVE EXPRESSIONS?

Most responses to the expressivist objection as applied to selective abortion maintain that there is some respect in which selective abortion cannot really be said to express a discriminatory or offensive judgment. There are several ways of making out this type of response, which could be given in defense of MAID, too, *mutatis mutandis*. Some argue that while the expressivist objection requires actions like selective abortion to be understood as communications, that is, as having symbolic or linguistic *meaning*, it is unlikely that they really *are* communications. Eva Feder Kittay, for instance, writes that

If many persons choose to abort fetuses with a particular characteristic, it is fair to make a hypothesis that those characteristics are devalued. But that is again, at best, a conclusion hypothesized about the causal factors that lead to the abortion and not a message that is sent out by the abortion. (2000, 188)

Later, she similarly states,

No one can judge the choice of another in these cases based on what is at best a degraded form of communication. No one can make a moral evaluation based on this incomplete communicative situation. . . to selectively abort because the fetus I carry is likely to develop into a child with disabilities does not send any clear and unambiguous message. (Kittay, 2000, 190)

Similarly, James Lindemann Nelson argued that the expressivist objection does not apply to most individual acts of selective abortion because the motivations behind them are multiple and unclear, the act itself is rarely *intended* as a form of communication and is usually not underwritten by a mutually-agreed-upon public system of symbols (Nelson, 2000).

Rather differently, Alan Buchanan (1996) and Bonnie Steinbock (2000) accept that selective abortion can have expressive significance, but think the expressivist objection still fails because most decisions to abort a fetus with a disability do not express beliefs of the kind needed for offense: those holding that persons with the disability should *never have been born*, or that *their lives have no value*. Selective abortion, they think, can express judgments about disabilities, but they are generally not the type of judgment it is reasonable to find offensive.

Another way of defusing the expressivist objection—though to my knowledge not well described in the literature—would simply be to deny that an individual decision to pursue MAID has *anything* to do with (other) disabled persons. Proponents of MAID could say that if a person requests MAID because she has some disability, no general judgment relevant to *other* persons with that disability is involved. For instance, one might say that even though a MAID requestor thinks it would be better to be dead than to live with quadriplegia, she is not thereby committed to thinking that others with quadriplegia would also be better off dead. In this view, our judgments about what is good for ourselves are radically parochial: they simply do not say anything about anyone else (Kim, 2022).

## IV. DEFENDING THE EXPRESSIVIST OBJECTION

My sense is that conventional responses to the expressivist objection fall short. Philip Reed argues briefly that attempts to defuse the expressivist objection are inadequate in the context of MAID:

Assisted death is almost always chosen while holding the belief that life is not worth living (or would not be worth living) under the condition of one’s illness and associated loss of capabilities. This is not merely a background belief, but almost invariably plays a role in the decision to die. . . . If the grounds for her believing that her life is not worth living involve her loss of capabilities, as we have seen that it almost always does for patients who choose to die, then the negative judgment towards life with a disability plays a role in her choice and so is more likely to express a message of disrespect. (2020, 540)

I am inclined to agree with Reed. Kittay and Nelson are too demanding in supposing that the expressivist objection needs selective abortion or MAID to be an (intentional) act of *communication*—all that the expressivist objection requires is that selective abortion or MAID have communicative significance,^1^ even if they are done for other reasons and any communication is unintentional. Admittedly, it is often unclear what an act expresses, and when it is unclear, the expression’s contribution to the moral valence of the act will be indeterminate. However, the expressive significance of acts that are not intentional communication is often very clear. The expressive significance of an act is often what it reveals about a person’s *values*—their desires, goals, priorities, wishes, and evaluative beliefs (i.e., beliefs of the form “this is good” or “that is disgusting”). If someone looks disgusted and gags when gazing at another person’s festering wound, it is clear that they are expressing disgust, even if this is not intentional and not an act of (symbolic) communication. Or for a subtler example, imagine that a father has two children of similar ages and brings a gift home, after a long trip, to only one of them. The overlooked child will undoubtedly feel hurt, since (barring some special story) he plausibly infers that his father was thinking more of his sibling. The father may not have intended to express or communicate anything at all. Yet, his act *did* express something and is morally criticizable because of what it expresses. Social creatures that we are, we frequently make inferences about what other people want and believe from their behavior; for a lot of the time, these inferences are correct.

Although Buchanan and Steinbock accept, *contra* Kittay and Nelson, that actions like selective abortion can have expressive significance, they place unduly narrow constraints on what kinds of expressions could be offensive. They assume that to be offensive to persons with a disability, judgments have to be of the form *persons with condition X, in virtue of X, have no claim to moral consideration* (or similarly: *they do not count in a moral sense*, or *they do not deserve to exist*, or *they ought never to have been born*,^2^ or *they lack moral status*). Let us call judgments that link an individual’s moral status to one of her characteristics *Form 1.* Judgments of this type lack what Stephen Darwall (1977) has called *recognition respect*.

There are, however, other forms of evaluative judgment that could underlie MAID chosen in response to a disabling condition—in general, these lack not recognition respect, but what Darwall (1977) calls *appraisal respect*. These different forms vary in multiple dimensions—in whether they are about the person or their characteristic, with respect to how universal they are, with respect to how responsive they are to intrinsic features of a disabling condition as opposed to its consequences, etc. Table 1 gives some examples of possible judgments that underwrite MAID in abstract form; it is not exhaustive, but at least it helps to distinguish judgments that might otherwise be regarded as identical.

**Table 1.** Example types of evaluative judgment that could underwrite a decision to request medical aid-in-dying

	Judgment Form	Judgment features
1	Persons with the disabling characteristic, in virtue of having that characteristic, have no (or reduced) claim to moral consideration	About the person Universal Related to intrinsic features of the disability
2	The disabling characteristic is itself so bad that if one has it one’s life is not worth living (it is better for the person with the characteristic to be dead or not exist than to live)	About the characteristic Universal Related to intrinsic features of the characteristic
3	The disabling characteristic makes it impossible to realize certain key values in one’s life, and these values are so important that if one cannot realize them one’s life is not worth living	About the characteristic Universal Related to typical or intrinsic consequences of the characteristic
4	The disabling characteristic makes it impossible to realize certain values in one’s life, and if one happens to have those values, it can be reasonable to prefer to die	About the characteristic Less universal (more dependent on personal values) Related to typical or intrinsic consequences of the characteristic
5	Because of some social circumstances, the disabling characteristic makes it very difficult to realize certain values, so if one happens to have those values, it can be reasonable to prefer to die	About the characteristic Less universal Related to consequences of characteristics contingent on social circumstances

Suppose a man requests MAID because he has developed the paralyzing condition amyotrophic lateral sclerosis (ALS). In principle, he *could* judge (Form 1) that individuals with ALS have no claim to moral consideration—that is, that they do not have the same (or any) claim to the rights and respect accorded to ordinary adult human beings (i.e., persons). But, he might think instead that ALS itself—the loss of movement capacities that it constitutes—makes one’s life no longer worth living (i.e., good for the person living it), irrespective of what one might value (Form 2). Less strongly, he could judge that it is reasonable to want to die if one has ALS, given certain values, *and* that most people should hold those values (Form 3). Instead, he might think that *because* he values certain things (like participating in sports or independence), it is reasonable for him to prefer to die than to live with ALS, while acknowledging that others need not have the same values (Form 4). Finally, he might think that, given the way his life is likely to go as he develops ALS, and primarily because of contingent social circumstances, it would be reasonable to prefer to die, given what he values, while recognizing that living with ALS might be better in different social circumstances (Form 5). There are undoubtedly other judgments that could underwrite his decision, but they probably fall between the different kinds of judgments illustrated above.

Elizabeth Barnes (2016) has distinguished between different ways we might judge that having a disability (or an injury or impairment that, in a social context, constitutes a disability) is a bad thing: we could judge either that it is *globally* bad, or that it is *locally* bad, or both. As I understand the distinction, it tracks two features of the disability and partially overlaps with the mapping of judgments I have laid out. The first feature is whether the impairment is itself bad (i.e., makes a person’s life less good, or makes them less well-off) relative to some normal state, or instead merely produces certain bad consequences (i.e., pain). The second is the degree to which the badness of the characteristic is contingent on (modifiable) features of the social milieu. Thus, one might think that dementia is bad in any of several respects: that the loss (or lack) of the cognitive abilities that constitute dementia is itself bad, that the intrinsic features of dementia also tend to produce bad consequences that, though not intrinsic, are still common (e.g., social isolation or anxiety), that the lack of cognitive abilities that is characteristic of dementia is bad itself in a way that is (or is not) contingent on the way our society happens to be, and that the consequences of dementia (like social isolation) either are or are not socially contingent.

Barnes argues that disabilities are (generally) not globally bad, but are at worst locally bad: she suggests that the “mere difference” view of disability is more plausible than the “bad difference” view. I do not engage, here, with whether Barnes is correct. What matters for my present purposes is whether the choice to pursue MAID is sometimes motivated by a judgment that a disability (or the incapacity in virtue of which the disability exists in a particular social setting) is globally bad or bad in itself, not whether such judgments are correct.

Buchanan and Steinbock implicitly concede that judgments of Form 1 should be offensive, which is plausible: what could be *more* offensive than someone judging that you do not have a right to exist because of what you are like? They also deny that judgments underpinning selective abortion (and, by extension, MAID) typically have this form. Even if we grant their second premise, however, they overlook that other forms of judgment from the spectrum outlined above might be reasonably^3^ offensive, too.

In other contexts, we are often justifiably offended by strong criticism that we regard as misinformed, unmerited, or unearned. Suppose someone (seriously) says that baldness is disgusting. Since I am bald, I could justifiably be offended, if only because the statement involves what strikes me as an excessively negative aesthetic evaluation of baldness. Worse, the critic might say that *bald persons* are disgusting. This could be at least slightly more offensive, presumably because it generalizes from one characteristic to an evaluation of the whole person who has that characteristic. It would be even more offensive if the critic said that bald men are so disgusting they should stay at home (or die). This involves an unreasonably negative, personalized evaluation, and also proposes a consequence of that evaluation that seems disproportionate and unearned. Generally, then, it might be reasonable to take offense at another person’s judgment if it involves blame, criticism, or reproof that seems unmerited, or a devaluation of things we think are valuable. The more we value the thing in question, or the more intense the criticism of what we value, the more serious our offense is likely to be.

Like judgments of Form 1, judgments of Forms 2 and 3 can be reasonably offensive. Judgments of Form 2 involve strong, potentially contentious evaluations of a personal characteristic and equally strong claims about what those evaluations mean you should do. Judgments of Form 3, although weaker than Form 2, are also potentially offensive, because they involve strong assertions about what persons should value. I take it that judgments of Form 4 are less likely to be offensive than Form 3 since they acknowledge that the value judgments that might underwrite MAID are not universal—but imagine that some could take offense simply at the fact that others devalue what they value. Form 5 judgments are the least likely to be offensive because they acknowledge that the values underwriting MAID are not universal and are focused primarily on the way social conditions affect a person, not on the characteristics of the person herself.

I suspect that MAID requests at least sometimes—perhaps typically—involve judgments of Forms 2 or 3, as opposed to Forms 4 or 5, or even Form 1. The main reason is that we often, across contexts, take ourselves to have reasons for acting that are not merely instrumental. Yes, we sometimes see ourselves as making choices on the basis of mere preferences: these are likings, dislikings, inclinations, and disinclinations that we regard as not dependent on reasons. One might choose a particular color of the car, for instance, just because one happens to like some options more than others, and regard oneself as having no other reason to do so. While this is a trivial choice, many of our evaluations and choices, especially those pertaining to weighty matters like life and death, at least seem to us to be reason-dependent. Now they may ultimately be linked to mere preferences psychologically (perhaps ultimate reasons can be given for none of our values), but we at least assume that others who are similarly positioned should want similar things and make similar choices.

To illustrate, consider the claim that one should not smoke: typically, this is underwritten by judgments along the lines that the reasons that favor smoking (e.g., pleasure) are outweighed by the reasons against it (e.g., effects on health). If I decide not to smoke, I probably think not only that the negative health effects of smoking outweigh the pleasure obtained *for me*, but that others should reasonably reach the same conclusion. When I claim that I should not smoke, I am making a judgment (or multiple judgments) about what is good and bad for people, about what one should or should not do, generally. Again, these judgments are not merely universal in an instrumental sense; they are also universal in the sense that they imply that everyone or nearly everyone should value health more than the pleasures of smoking, at least given a certain set of background assumptions about what they are like.

Judgments about quality of life that motivate MAID are often similarly universal. If one says “I do not want to live with advanced ALS because it will undermine my quality of life substantially,” then, unless one’s declaration is clearly underwritten by what one regards as mere preferences, one may well be judging that others who are similarly situated should *reasonably* not want to live with ALS.^4^ One might even recognize that others disagree and would not make the same choice, but still think that they *should* make the same choice. Even if we accept that values are essentially contested, we are not thereby committed to thinking that they are purely relative.

To reinforce my point, note that it is unlikely that such a pronouncement about ALS is intended as a statement of *mere* preference: one is unlikely to hang such a controversial choice as MAID on justifications that are prima facie arbitrary. Consider how strange (and off-putting!) it would be to say, “I want to die, but for no particular reason; I just feel like it.” While one’s judgment about ALS, when one chooses MAID in the face of it, might allow exceptions for persons who are not similarly situated (one might concede that, if a person derived all his satisfaction from intellectual pursuits, then his quality of life would not be too severely impaired by ALS), it at least has implications for persons who are similarly situated—that is, for persons to whom similar reasons apply. To the extent that some persons with disabilities are in relevantly similar circumstances, the judgment of persons with ALS implies that they, too, should reasonably think that they are better off dead.

## V. IS IT WRONG TO OFFEND OTHERS?

To this point, I have argued that a request for MAID in the face of some medical condition often expresses underlying evaluative judgments that may apply to other persons with that (or a relevantly similar) condition, and that it can be reasonable for them to find those judgments offensive. For the patient-level expressivist objection to the matter, however, it must not only be true that requests for MAID have offensive expressive significance but also that all of us have strong obligations to avoid offending others in that way—that is, that private citizens should refrain from acting on “ableist” values *because* such values are offensive to persons who are disabled when they become aware of them. Other responses to the expressivist objection take this for granted, but we should not.

What moral weight should we attribute to the fact that a person is offended? Joel Feinberg, though focused on defining the appropriate scope of criminal law, provides the most extensive account of this topic. Feinberg (1985) claims that a person is offended when she suffers a disliked mental state that she attributes to the conduct of another person, and she resents that other person for his role in causing the disliked mental state. Feinberg (1985) suggests that being offended is distinct from and generally less serious than being *harmed* so that our strong obligations to prevent harm to others do not automatically apply to offense. Feinberg (1985) also claims that we should evaluate whether offensive conduct should be prohibited by considering several criteria: the value of the offensive conduct, whether it is innocent or spiteful (especially whether it is *intended* to offend), whether those who are offended could easily avoid exposure to the offending act, and the seriousness of the offended mental state itself (a function of its intensity, duration, and extent). Criteria like these are necessary to limit abuses of the law by those who are too readily offended. People are offended by all sorts of things, and if we prohibited activities merely because they might be offensive to someone, we would have to prohibit most activities.

It would be tempting to help ourselves to Feinberg’s distinction between offense and harm in replying to the expressivist objection, and simply say that the offense that persons with disabilities might take from others’ decision to pursue MAID, since it is not a harm, is not so morally significant that MAID should be prohibited. This is not sufficient, however, for several reasons. First, I doubt that Feinberg’s distinction between harm and offense is correct. Indeed, we can quickly make a compelling case that being offended *is* a harm, though often not a very serious or lasting one. I have at least a weak interest in not experiencing negative, unpleasant emotional states, simply because they are unpleasant. If I have to choose between two actions, A and B, and they have the same features except that B makes me sad, my interest in not being sad is arguably a sufficient reason to choose A over B. Because undermining an interest constitutes harm, so making me sad is a harm, if only a small one. Likewise, it is often morally wrong for me intentionally to bring about negative emotional states in others; for example, it would be wrong for me to terrify others (without their knowledge or consent) simply for my own amusement; in contrast, it is often not wrong for me to amuse others through humor without their consent.

Second, it would be easy to underestimate the emotional significance of some kinds of offense. When offensive conduct is directed at one’s *self* or one’s *identity,* it can be *very* emotionally painful. It can also occasion serious interpersonal conflict (consider how common it was, in some cultures, to defend one’s honor through violence). What others think of us can matter a great deal. This is not even to consider the fact that both acute and chronic distress have been shown to have negative effects on physical health (Hughes et al., 2017; Shonkoff, Slopen, and Williams, 2021); the claim here is that to inflict emotional distress *itself* is to harm.

Along similar lines, consider that at least three factors could aggravate the offensiveness of MAID for persons with disabilities. First, they are highly likely already to be the victims of offensive conduct elsewhere—this, if the emotional strains of offense are cumulative, could make offense from MAID more acute. Second, persons with disabilities may tend to be among the least well-off in a society, such as ours, where resources are distributed unequally and accommodations for disability are often insufficient. This may mean, following something like Rawls’ (1971) difference principle, that we have an extra obligation to assure that they are not exposed to other costs from our actions. Third, because persons with disabilities may either identify as such, or (what I take to be the same) identify with their disabilities, the offended states they experience related to acts that devalue disability may be especially intense.

While these aggravating factors are considerable, and while the offensiveness of MAID may sometimes constitute harm, the kinds of conduct that are impermissible because they are offensive to persons with disabilities still need to be determined in the way Feinberg describes. Feinberg’s criteria are needed because, again, it would be unreasonable to suppose that any act that might offend a person with a disability, which seems to be related to their disability per se, is therein morally wrong.

To illustrate this last point, suppose that Alex, at age 22, sustains a serious spinal cord injury while surfing in Hawaii. The injury causes quadriplegia. Imagine that, after a period of bitterness and emotional turmoil, he accommodates to his injury and even comes to identify as someone with quadriplegia. Despite his disability, he has a life he regards as fulfilling: he is a successful computer game designer, lives in an area where his mobility (aided by a motorized wheelchair) is not unduly limited by structural barriers, and has many social and recreational outlets: music, romance, close friends, etc. During a family reunion in Fiji, however, Alex observes that his 22-year-old cousin, Mark—usually an avid surfer—has eschewed the opportunity to go surfing at the beach in front of their hotel. His suspicions heightened, and Alex confronts Mark, who reveals that he chose to refrain from surfing because he has been visually confronted by Alex’s injury and does not want the same fate to befall him. Alex is offended: how dare Mark imply that his life is not a good one, that his paralysis is a bad thing?

We can understand Alex’s feelings. We might even think Mark is obligated to respond to them in some respect—that is, by apologizing. Yet, does Alex’s being offended entail that Mark is obligated to go surfing or that he committed a moral wrong when he refrained from surfing? It does not seem so. Alex’s being offended, though morally important, is not so weighty as to make some of Mark’s otherwise self-regarding activities (things he does that affect his own interests much more than others’ interests) impermissible. It would, of course, be different if Mark decided to walk around in a neck brace simply to ridicule Alex—then his conduct would be wrong, but mainly because it was spitefully intended to offend.

One might draw a somewhat different conclusion about cases like this. Reed notes that there is an important difference between what he calls “existential” methods of reducing disability and “non-existential” methods of doing so—the former involves preventing or ending the lives of persons with disabilities, and the latter leaves the persons themselves alone and affect the disability only (2020, 543). Reed suggests that existential methods convey negative judgments against disabled *individuals* while non-existential methods only convey negative judgments about disabilities themselves, not about the individuals who have them. Thus, Mark’s steps to avoid injuring himself express the disvalue of quadriplegia itself, not of Alex himself or other persons with quadriplegia (Alex expresses appraisal disrespect but not recognition disrespect) and so should not be seen as offensive or disrespectful.

I am not convinced by this response, largely because non-existential methods can also be offensive: they clearly convey judgments about characteristics (as Reed would agree), and, as I argued earlier, judgments about characteristics themselves can be offensive; it is not necessary that the disvaluing judgment be about the individual himself.^5^

We might, then, doubt that private citizens should, *as a rule,* have to refrain from engaging in various kinds of conduct because they are offensive to persons with disabilities (or anyone else, for that matter), where such conduct is itself at least somewhat valuable to them and not spitefully aimed at giving offense. This can be true, even if the characteristics that are implicitly devalued through that conduct are identity-defining. These characteristics might be sufficient to distinguish MAID from clearly wrongful offensive practices, such as using ableist, discriminatory epithets: this is something that people generally do not have any independent interest in doing, and is most often intended to offend, so the offended feelings it occasions are almost always decisive reasons against it (for more on this, see my remarks about hate speech in Section VI).

A balancing of competing interests is an essential part of determining whether offensive conduct is wrong: something that imposes enormous emotional distress on an already disadvantaged person in exchange for advancing a very trivial interest is much more likely to be impermissible than an action that advances a very important interest at the cost of a small zephyr of distress in someone who is otherwise fully advantaged. This balancing of interests requires that we consider the decision to engage in the conduct both instrumentally—whether it is necessary for advancing the goal it is intended to advance, given the alternatives—and consider the importance of the goal attained relative to the offended feelings involved. We should then note that, whereas species of offensive conduct that are clearly morally wrong are generally motivated by bad or unimportant goals and further corrupted by misinformation, MAID is usually motivated by values and beliefs that are reasonable if still contentious and is often plausibly taken to be the best—indeed, the only—way of achieving those goals. In short, it is plausible that the interests at stake for persons who request MAID to alleviate their own pain, existential distress, and despair at their lack of independence outweigh any of the interests *other* persons who have disabilities might have in not being offended.

My point is not that we can never have obligations to avoid offending others, whether they have a disability or not. To the extent that others would have difficulty avoiding knowledge of our conduct, how they feel about that conduct contributes to whether it would be wrong. My point is, instead, that we should not assume that the bare fact that others could reasonably find our conduct offensive means that we should not do it.

## VI. OBJECTIONS

I want briefly to consider some objections to my analysis of patient-level expressions. First, one might object that the choice to pursue MAID is at least sometimes motivated by judgments that are about the value of persons themselves (like Form 1), not about their characteristics. If so, the offensiveness of MAID might be great enough to make it more broadly impermissible: while it might be permissible to regard other persons’ quality of life as unacceptably bad, it is not permissible to insist that they are not persons.

I doubt, however, that judgments like Form 1 can often be attributed to persons who choose MAID for themselves, largely because it is implausible to think that such persons often treat themselves as lacking moral claims in other respects (and when they do, this is often seen as a sign of psychopathology, especially severe depression). But, the objection might be reframed: because it is *sometimes* the case (or merely *possible*) that persons would choose MAID for deeply problematic reasons like Form 1, the practice generally is morally impermissible.

We should reject this conclusion. First, note that there are two issues at stake: whether, because some persons might choose MAID for deeply unpalatable reasons, the practice as a whole should (as a matter of law, perhaps) be prohibited, and whether individual MAID choices could be wrong regardless of the reasons behind them because some MAID choices might be motivated by bad reasons. Regarding the first issue, however, it seems clear that we should not generally prohibit a practice because some persons might engage in that practice for bad (e.g., discriminatory) reasons. For instance, it could be that a few persons in our society want to wear red baseball caps because it signals allegiance to a racist, nationalist political organization; nevertheless, most others who wear red baseball caps do so for innocuous reasons, and thus the practice should be permitted. Regarding the second issue, there seems to be no principled reason we treat all MAID choices as the same: we can instead say that some MAID choices are morally wrong because of their reasons, while others are morally permissible.

A different problem arises if Barnes (2016) is correct that disabilities are *mere differences* rather than *bad differences*—for if a person chooses to end her life rather than succumb to some disability because she thinks that disability is bad *in itself*, but she is mistaken, then her interest in acting this way may be much diminished, and so less able to counterbalance the interests of the others whom she offends. Note, too, that the observation that people often habituate to a disability can play the same role in thinking about the expressivist objection since it would also imply that a person’s reasons for pursuing MAID are not particularly good.

It is not possible to evaluate Barnes’ arguments here, though I say briefly that I see her as primarily doing two things: (1) casting doubt on claims that disabilities are bad differences, and (2) arguing that we should take seriously the assertions by persons with their disabilities that they do not see their disabilities as bad. This still, however, leaves open the possibility that the value and disvalue of many disabilities is an essentially contested matter. In that case, if a person believes that her disability is a bad difference, we should also see her judgment as having local authority. In this sense, the judgment that a given disability is bad is much like the judgment that one should adhere to a particular religion—to the extent that compelling arguments cannot be made for any given view, we can do no better than to accept that there is disagreement.

## VII. STATE-LEVEL EXPRESSIONS

At the state level, whether MAID laws express offensive evaluations of persons with disabilities depends on how they are structured. If the state simply refrains from restricting MAID for autonomous persons (as is approximately the case in Switzerland), then the permission *itself* cannot plausibly be seen to express anything offensive; it eschews all judgments about citizens’ reasons for action. At worst, this regime could offend because it fails to prohibit citizens from acting on their own offensive judgments.

If, however, MAID is permitted in some situations but restricted in others, then the state *itself* seems to express potentially offensive evaluations about persons’ lives, as Kim (2022) and others have noted. In the United States, for instance, the 11 jurisdictions that permit MAID allow it only for persons with terminal illnesses (Compassion and Choices, 2022); this implies a position that MAID is reasonable for persons with terminal illnesses but not others. Similarly, jurisdictions (like the Netherlands) that permit MAID for persons experiencing severe suffering or incapacity related to illness (even if not terminal) seem to express the state’s judgment that suffering or incapacity can make a person’s life not worth living (Dyer, White, and Rada, 2015).

Should the state prohibit MAID because what it would express by permitting it is (or could be) offensive to some of its citizens? It is clearly not sufficient to claim that the state should never do things that offend its citizens: this would have ridiculous consequences, such as that the state could not permit marriage between persons of two different religions or races because it offends some other citizens. Proponents of the expressivist objection must therefore link state-level offensiveness of MAID to some of the state’s other obligations. There are two main ways to do this, both Rawlsian and not strictly Rawls’. First, they might claim that the state should be neutral with respect to its citizens’ different (reasonable) conceptions of the good, which are roughly their ideas about what makes for a good life and any associated goals, plans, etc. Call this idea *state neutrality*. Under state neutrality, the state treats its citizens as equally able to choose what is good for themselves. Instead, MAID opponents might claim that the state should treat all of its citizens as moral equals—that is, as having equal claim to its support and protection. Let us call this idea *equal regard*.^6^ Permitting MAID for persons with a terminal illness or severe disability (but not otherwise), they might claim, violates either or both of these requirements, justifying the offense that persons with disabilities feel.

Neither approach is successful, however. Consider state neutrality first. It has at least two forms, one thin, and one thick. On the thin form, neutrality means that the state must refrain from prohibiting the pursuit of different conceptions of the good if the actions involved are ostensibly self-regarding. Specifically, the state should not prohibit actions on the basis of its judgment that the conceptions of the good motivating them are incorrect or flawed; it should not, at least beyond some minimal criteria of reasonableness, evaluate conceptions of the good. For instance, thin neutrality means that the state cannot prohibit the practice of some religions (where worshipping in a certain way can be an important part of someone’s conception of the good) because it judges those religions to be less good or correct than competitors.

The thin version of state neutrality certainly makes trouble for selective MAID regimes, but not the kind of trouble proponents of the expressivist objection want. Because these regimes prohibit MAID done for some reasons but not others, they necessarily violate thin neutrality. But thin neutrality only supports the expressivist objection if the state *prohibits* self-regarding acts motivated by conceptions of the good that view disabilities positively. On thin neutrality, one has grounds for complaint only if the pursuit of one’s own conception of the good is prohibited; but under limited MAID regimes, disability-positive conceptions of the good are neither valued nor devalued.

To explain further: by permitting persons to act from conceptions of the good that devalue disability, the state is not actually failing to be neutral with respect to disability-positive conceptions of the good. Thin neutrality means that the state cannot *endorse* some conceptions of the good in favor of others, but it does not—and *cannot*—prevent the state from *accepting* different competing conceptions. Acceptance and endorsement are different. If the state does not *prohibit* self-regarding actions required by a particular conception of the good, then it implicitly accepts that conception of the good as legitimate. Endorsement, in contrast, means evaluating a conception of the good more positively than competitors; this means either actively promoting that conception of the good or impeding the pursuit of its competitors. If the state permits MAID in the face of severe disability, then it accepts conceptions of the good according to which severe disability makes a life go worse. But, this is compatible with *also* accepting disability-positive conceptions of the good. Indeed, thin neutrality is only achieved if the state accepts all competing reasonable conceptions.

The thick version of state neutrality pertains to the allocation of social goods and adds to thin neutrality the requirement that the state should not distribute social goods in ways that advantage some conceptions of the good and disadvantage others.^7^ For example, it entails that the state should not adopt policies that make certain religions more difficult to practice than others—for instance, by distributing land or subsidies to one religious organization but not to others.

Even if we accept thick neutrality, however, it would not necessarily support the state-level objection, since simply permitting MAID does not entail much in the way of distributing resources; thick neutrality would ground an expressivist objection only if MAID were largely financed by the state *and* if it were enacted to the exclusion of programs that benefit persons with disabilities. Even where the state agrees to pay for MAID, this violates neutrality only if the state fails to devote adequate resources to the realization of disability-positive conceptions of the good. To the extent that it does fail in this way, the right response is clearly not to prohibit MAID. It would seem better to shore up the resources devoted to persons with disabilities.

What about equal regard? One might interpret the state-level expressivist objection as saying that permitting MAID entails treating certain citizens—those with disabilities—as less worthy of respect (and thus as not equal); as Sulmasy et al. (2018) said, MAID seems to impugn the dignity of persons with disabilities. After all, to permit MAID under the usual restrictive criteria implies that it can be reasonable to kill oneself because of the disadvantages associated with some life-limiting illness or injury. It is no great stretch to imagine that this involves seeing lives marked by such disadvantages as worse.

In permitting MAID—even if only in limited circumstances—the state does not necessarily imply anything about the moral worth of its citizens. Suppose, to consider the hardest case, that some citizens are motivated to pursue MAID by judgments of Form 1—that is, in virtue of their conditions, they think their own lives are no longer worth living and that they have reduced moral claims. The state could permit these citizens to treat themselves in ways that reflect their Form 1 judgments while ensuring equal regard, so long as it remained willing to continue to treat those persons as having full moral claims, giving them equal access to resources and protections if they wish. The state could while striving to treat all citizens equally, coherently permit persons to treat themselves as unequal, at least in some respects. Again, the state’s implicit acceptance of some conception of the good does not imply endorsement of it.

One might be tempted to couch the concern about equal regard in terms of the state’s obligation to ensure what Rawls (1971) called the *social bases of self-respect*. The state provides its citizens with the social bases of self-respect if it treats them justly and also transparently communicates to them that it is doing so *because* it sees them as morally equal. A well-ordered society, Rawls (1993) says, is one where citizens adhere to the basic principles of justice and satisfy a publicity requirement: they know that others adhere to the principles of justice, too, and know that others know this about them in turn. It might be suggested that the state fails to provide the social bases of self-respect for disabled citizens when it permits (limited) MAID, simply because it fails to *prove* to them that it is showing equal regard. Even if it otherwise succeeds in treating them as equals, it fails to ensure the social bases of self-respect simply by tolerating the appearance that it does not treat them equally.

The weight of the expressivist objection understood this way, falls on the publicity requirement. In general, the publicity requirement does not entail anything about what the state should do *apart* from what it should say about its motives and justifications. That is, the fact that citizens might mistakenly (but still plausibly, in the absence of careful philosophical analysis) interpret MAID policy as showing a failure of equal regard does not itself make the policy wrong; it only means that the state needs to do a better job of explaining itself. For comparison, consider a similar point about vaccine mandates: if a vaccine mandate is otherwise justified, but a substantial minority of citizens mistakenly regard it as unjust, the appropriate response is not to overturn the mandate; it is to try to convince the public of its justification.

This leaves us with a question: in the interest of demonstrating equal regard, should the state prohibit certain actions by individual citizens because they betray individual judgments that might be offensive to others? Imagine again that the state simply does not prohibit citizens from accessing MAID; some will surely access it because they subscribe to judgments of Forms 1, 2, or 3. Does the state do wrong simply because it fails to prohibit actions motivated by such offensive judgments? Here we can turn back to Feinberg, whose answer would clearly be *no*: at most, the offense occasioned by an action has to be considered against its other benefits and harms, relative to alternative actions, before we decide to prohibit the action. We might, as an added consideration, worry that prohibiting actions because they are offensive would probably violate thin neutrality since the state would be prohibiting actions on the grounds that other citizens do not like the conceptions of the good underwriting them.

Consider, too, that more stringent prohibitions on (otherwise) self-regarding actions because of their expressive significance would be unappealing, especially if directed at actions motivated by judgments of Forms 2 and 3. Just as actions that express negative evaluations of disability are not necessarily morally wrong for that reason (as with the case of Alex and Mark in Section V), the state cannot reasonably prohibit people from many acts other than MAID that express negative evaluations. People might, for instance, wish to wear helmets while biking, largely to reduce the risk that they will have a head injury and become disabled. Others might also wish to purchase a house that is free from lead paint since they know that exposure to lead paint can lead to intellectual disability and want to avoid that for their children. Though less acutely than MAID, each of these choices itself entails a negative evaluation of certain kinds of disability—and yet should be permitted.

This is not to say that the state cannot restrict *any* actions that express negative evaluations of other groups. Restrictions on hate speech, for instance, look justified. Jeremy Waldron defends them on the grounds that hate speech and related expressive acts (for instance, publishing defamatory claims about persons from some ethnic group, burning a cross, painting swastikas on a synagogue, etc.) threaten the dignity of the citizens they target, not primarily because they express hate itself, but because they are intended to make social conditions less hospitable. In Waldron’s view, the point of hate speech laws is to

vindicate public order, not just by preempting violence, but by upholding against attack a shared sense of the basic elements of each person’s status, dignity, and reputation as a citizen or member of society in good standing—particularly against attacks predicated upon the characteristics of some particular social group. (2012, 47)

Waldron is at pains to point out, however, that prohibitions on hate speech are *not* justified by considerations of offense:

A dignitarian rationale for legislation against hate speech or group defamation differs from an approach based on the offense that may be taken by the members of a group against some criticism or attack. . . . The distinction is in large part between objective or social aspects of a person’s standing in society, on the one hand, and subjective aspects of feeling, including hurt, shock, and anger, on the other. A person’s dignity or reputation has to do with how things are with respect to them in society, not with how things feel to them. (2012, 106)

Indeed, Waldron is concerned that legislation designed to prevent offense would have unsustainable consequences:

I believe that Jesus Christ is the Son of God. . . and of course my right to believe that is one of the core interests that must be protected in society come what may. But can I plausibly demand—in the same non-negotiable spirit—a social environment in which this view is never contradicted or made fun of? Of course not. Many other creedal claims, held as fervently as mine, deny this belief about Jesus. . . I may be distressed by these denials. . . [b]ut I have no right to demand the suppression of these views on the ground that they offend me. (2012, 135)

A proponent of the expressivist objection might claim that MAID could be prohibited as a sort of hate speech (with “speech” defined broadly). But we should not accept this. Note that in deciding whether to prohibit a practice on the grounds that it is hate speech, the state has to make some informed guesses about why people are engaged in it. Displaying a swastika is generally done by anti-Semitic groups to terrorize Jews, so it should, in Waldron’s view, be banned; but the swastika (typically, but not always, reversed) can also be displayed as part of sincere Hindu religious observances that have no anti-Semitic import—in which case, it should not be regarded as hate speech. Prosecution of persons who display swastikas, therefore, should attend to evidence of their motives.

Now consider again my distinction between the forms of judgment that can underpin MAID requests. Only judgments of Form 1 could plausibly be understood as impugning the dignity of persons with disabilities in Waldron’s sense; judgments of Forms 2–5 do not. If it is true that Form 1 judgments rarely underwrite MAID, then the case for prohibiting MAID as hate speech fails at the outset. But there is a second requirement in Waldron’s view to consider. To count as hate speech, an act must do more than simply express the judgment that persons of a certain group lack dignity or moral status: it must also be intended to propagate that view within a society and to make conditions in that society less welcoming for members of the group. Even MAID choices motivated by Form 1 judgments presumably do not have that intention, so they should not be regarded as hate speech.

## VIII. PROVIDER-LEVEL EXPRESSIONS

I now offer only a few quick words about the expressivist objection and medical providers. To this point, I take myself to have shown that the expressivist objection is unpersuasive at both the individual and state levels. Medical providers fall somewhere between: they are invested with state authority, so they are partially agents of the state, but they are also given wide discretion to apply their own evaluations in their work. It is plausible that, given this middling position, medical providers would be bound by the expressivist objection only if it held at either the individual or the state level; since it holds at neither level, we might conclude that they do not have any strong obligation to avoid offending persons with disabilities by providing MAID. It is conceivable that physicians’ specific professional obligations (perhaps deriving from the Hippocratic Oath or some similar code) would create independent requirements to avoid giving offense. Such obligations are unlikely to be uncovered since there are many other respects in which physicians *already* permissibly make expressions that (as with patients) convey a devaluation of disability: a physician can, for example, agree to try saving an injured leg while refusing to amputate a healthy leg—therein expressing the judgment that being able to walk is better than being unable to. This could be offensive to persons who cannot walk, but this does not make it impermissible.

## IX. CONCLUSION

In considering the permissibility of MAID and how we think about disability, it is important to bear in mind that most people who request and receive MAID are, in a way, desperate. In the United States, they are usually facing a terminal medical condition; even countries that do not require a terminal diagnosis often still require that MAID recipients suffer severely and irremediably. Although proponents of the expressivist objection are probably correct that a patient’s decision to receive MAID expresses a negative evaluation of his disability, and although it could be reasonable for others with that disability to be offended by this, it is not obvious that this offense makes MAID morally wrong, nor that the state has some obligation to prevent that offense, nor that the state, if it offends him in permitting MAID, acts wrongly. A pluralistic society, where there are many competing and antithetical conceptions of the good, and where we strive to make the maximum amount of space for people to realize their own conceptions of the good, is also one in which a fair amount of offense will probably occur. Although we may feel pressure to regard all conceivable ways of being as equally worthy of approval, and sometimes pretend that we think they are, in reality, we often have strong views about how we, and others, should live. The only coherent pluralism is one that, while demanding that we see others as having intrinsic moral worth, permits us to think that their values are wrong, and sometimes even that their lives are less good.

## Footnotes

1 As an anonymous reviewer pointed out, the expressive significance of selective abortion and MAID may not be entirely equivalent; selective abortion because the decision is often dominated by one fact about the fetus, can put disability in the foreground more than MAID, which can reflect many different aspects of a person’s life. Still, this does not preclude MAID from having expressive significance relevant to persons with disabilities.

2 Note that there are two different ways one can interpret claims like “X ought never to have been born.” One interpretation has this as very close to “X does not *deserve* to exist”—meaning that, because of his nature or characteristics, X has no or greatly reduced moral status and should not presume to lay claims to the goods of citizenship. The other way of understanding the phrase is purely prudential: it is to say that X ought never to have been born, even though X *does* deserve moral consideration because life is overwhelmingly bad for X. My suspicion is that many who write about the expressivist objection on both sides run these two interpretations together. Using the classification I laid out, this is basically a matter of conflating judgments of Form 2 with judgments of Form 1.

3 Any judgment could be offensive in practice, but the offense can be more or less reasonable. It is reasonable for you to be offended if I ridicule your religion; but not reasonable for you to be offended (absent some very special story) by the fact that I ate a grape last Tuesday.

4 It is nevertheless true that we accept a sort of modest pluralism about values. Most of us recognize that people value different things as a matter of fact. I suspect most of us also regard an array of different and conflicting values as reasonable. We can accept, for example, that some people want to have children, and that some people do not, and see both values as reasonable. But we also clearly do not regard all values as reasonable. We tend to see health as something most people should want. If someone takes risks with his health without good reasons—suppose, for instance, that he has been playing with radium liquid, putting it on his face and hands, writing his name on the table with it, and so on because he thinks the way it glows in the dark looks nice, even though he *knows* that it is radioactive and could cause cancer—we will think that he is simply wrong to prioritize these temporary (and relatively crass) amusements over his health. Elsewhere (Kious, 2016) I have argued that such comparative judgments about a person’s values are inescapable if we are to engage in moral reasoning; here, however, all that need be assumed is that our thoughts about values have this form.

5 Of course, I would also say that Reed errs in supposing that existential methods say something about the worth of the person—they can do so but typically do not.

6 It is clear that most modern states fail to treat persons with disabilities as moral equals in many respects, but the question here is whether they fail to do so by permitting MAID.

7 It is worth noting that it might be difficult, in practice, to spell out exactly what this means in ways that would allow the state ever to devote its resources to promoting certain goals or values, such as health or education.
